# At a Supra-Physiological Concentration, Human Sexual Hormones Act as Quorum-Sensing Inhibitors

**DOI:** 10.1371/journal.pone.0083564

**Published:** 2013-12-23

**Authors:** Amélie Beury-Cirou, Mélanie Tannières, Corinne Minard, Laurent Soulère, Tsiry Rasamiravaka, Robert H. Dodd, Yves Queneau, Yves Dessaux, Catherine Guillou, Olivier M. Vandeputte, Denis Faure

**Affiliations:** 1 Institut des Sciences du Végétal (ISV) UPR 2355, Centre National de la Recherche Scientifique, Gif-sur-Yvette, France; 2 Seeds Innovation Protection Research and Environment (SIPRE), Comité Nord Plants de Pommes de Terre (CNPPT), Achicourt, France; 3 Institut de Chimie des Substances Naturelles (ICSN) UPR2301, Centre National de la Recherche Scientifique, Gif-sur-Yvette, France; 4 Institut de Chimie et Biochimie Moléculaires et Supramoléculaires (ICBMS) UMR 5246, INSA Lyon-Université Lyon 1, Villeurbanne, France; 5 Laboratoire de Biotechnologie Végétale, Université Libre de Bruxelles, Gosselies, Belgium; Ghent University, Belgium

## Abstract

N-Acylhomoserine lactone (AHL)-mediated quorum-sensing (QS) regulates virulence functions in plant and animal pathogens such as *Agrobacterium tumefaciens* and *Pseudomonas aeruginosa*. A chemolibrary of more than 3500 compounds was screened using two bacterial AHL-biosensors to identify QS-inhibitors (QSIs). The purity and structure of 15 QSIs selected through this screening were verified using HPLC MS/MS tools and their activity tested on the *A. tumefaciens and P. aeruginosa* bacterial models. The IC_50_ value of the identified QSIs ranged from 2.5 to 90 µg/ml, values that are in the same range as those reported for the previously identified QSI 4-nitropyridine-N-oxide (IC_50_ 24 µg/ml). Under the tested culture conditions, most of the identified QSIs did not exhibit bacteriostatic or bactericidal activities. One third of the tested QSIs, including the plant compound hordenine and the human sexual hormone estrone, decreased the frequency of the QS-regulated horizontal transfer of the tumor-inducing (Ti) plasmid in *A. tumefaciens*. Hordenine, estrone as well as its structural relatives estriol and estradiol, also decreased AHL accumulation and the expression of six QS-regulated genes (*lasI*, *lasR*, *lasB*, *rhlI*, *rhlR*, and *rhlA*) in cultures of the opportunist pathogen *P. aeruginosa*. Moreover, the ectopic expression of the AHL-receptors RhlR and LasR of *P. aeruginosa* in *E. coli* showed that their gene-regulatory activity was affected by the QSIs. Finally, modeling of the structural interactions between the human hormones and AHL-receptors LasR of *P. aeruginosa* and TraR of *A. tumefaciens* confirmed the competitive binding capability of the human sexual hormones. This work indicates potential interferences between bacterial and eukaryotic hormonal communications.

## Introduction

Bacterial populations synthesize and exchange chemical signals which coordinate and synchronize gene expression in a cell-density dependent manner. Such regulatory pathways are called quorum-sensing (QS) and involve diverse QS-signals, including *N*-acylhomoserine lactones (AHLs) [1]. The canonical proteins required for the synthesis of AHLs belong to the LuxI family, and those for AHL-sensing to the LuxR family [2]. The AHL-mediated QS is widespread among Proteobacteria, controlling - for instance - the expression of genes involved in bacterial virulence in animal and plant hosts, horizontal gene transfer by plasmid conjugation, as well as bacterial competitiveness in the environment through production of antibiotics [1]–[2].

Natural and synthetic compounds which alter QS signalling and thereby disrupt QS-regulated gene expression are called QS inhibitors (QSIs). Considering the central role played by QS in the expression of virulence genes in pathogenic bacteria, the search for QSIs has driven many efforts [3]. Over the past several years, numerous QSIs with diverse structures have been identified using different approaches such as the synthesis of structural analogues, experimental and virtual screening of chemo-libraries and purification of natural QSIs from diverse organisms, especially plants [3]–[5]. The natural QSIs contribute to host defense against bacteria and both natural and synthetic QSIs have been proposed as promising molecules because they may act synergistically with antibiotics to limit bacterial infection [6]–[8].

In this work, we screened a chemo-library for the presence of QSIs and validated the QSI activity of the identified compounds using two bacterial species, the plant pathogen *Agrobacterium tumefaciens* in which QS regulates the horizontal transfer of the tumor-inducing (Ti) plasmid, and the opportunistic pathogen *Pseudomonas aeruginosa*, in which QS controls the expression of virulence factors. This paper reports the identification of novel natural (hordenine) and synthetic (indoline-2-carboxamides) QSIs, and also experimentally demonstrates QSI-activity of three human sexual hormones: estrone, estriol, and estradiol.

## Results

### Identification of the QSIs

**Figure 1:**
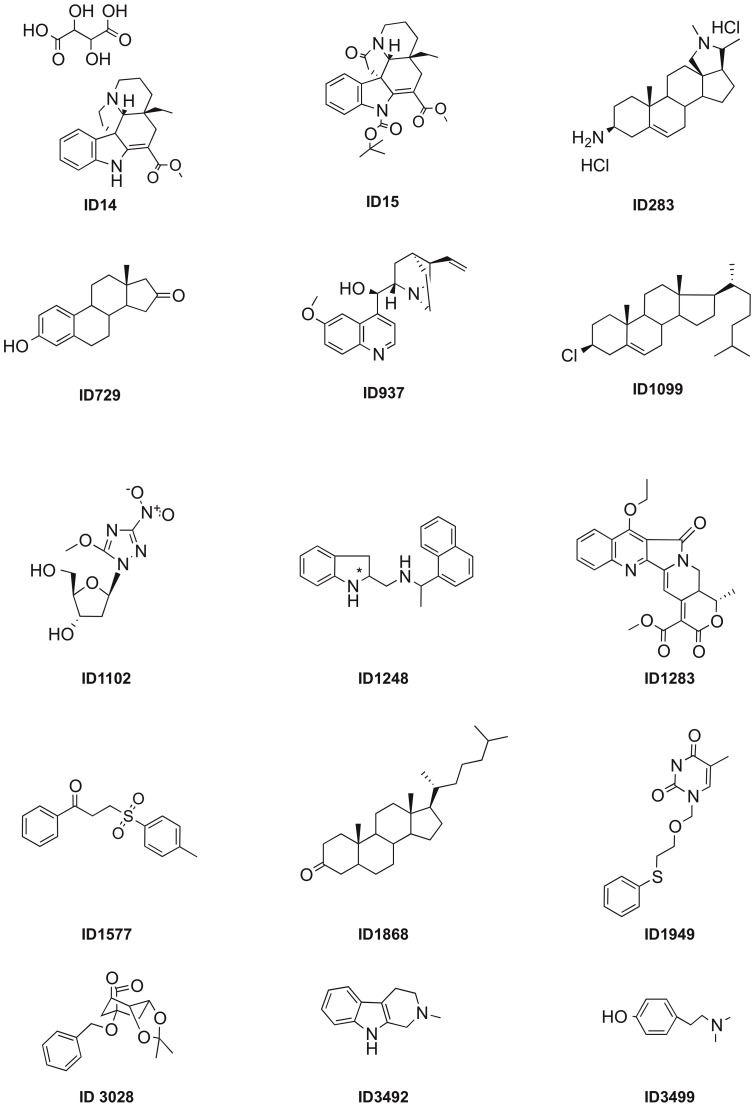
Structures of the QSIs identified in the chemical library.

The ICSN chemical library (see materials and methods) was screened with two bacterial AHL-bioindicators, *C. violaceum* CV026 and *A. tumefaciens* NT1(pZLR4) in the presence of the appropriate AHLs. The strains and plasmids used in this study are listed in Table 1. Using *C. violaceum* CV026 in association with hexanoylhomoserine lactone (C6-HSL) at 0.5 µM and the tested compounds at 50 µg/ml, over 150 potential QSIs corresponding to ca. 5% of the chemical library compounds, were identified. To improve the selectivity of the screening, we reduced the concentration of the tested compounds to 5 µg/ml and used the *A. tumefaciens* biosensor which is sensitive to very low amounts (10 nM) of octanoylhomoserine lactone (C8-HSL). From this second screening, 25 molecules, i.e. 0.7% of the 3520 tested compounds, emerged as potent QSIs. Ten out of the 25 identified molecules (e.g. novobiocin, quinine, ochrolifuanine A and o, β-dinitro-β-methylstyrene) are already known as antimicrobial agents. They were consequently removed from this study. Hence, only 15 of the identified hits numbered **14**, **15**, **283**, **729**, **937**, **1099**, **1102**, **1248**, **1283**, **1577**, **1868**, **1949**, **3028**, **3492**, and **3499** were retained for further analyses (Figure 1). Compounds **14** [9]–[10], **15** [11], **1102** [12], **1283** [13], **1577** [14]–[15], **3028** [16] have previously been described, while compounds **283**, **729**, **1248** and **1949** are described in the experimental section. Compounds **937**, **1099**, **1868**, **3492** and **3499** are commercially available (Sigma Aldrich and SynChem, Inc.). The 15 hits belong to different structural families such as carbazole (i.e. **15**), indoline (i.e. **1248**), pyridoindole (i.e. **3492**), steroids (i.e. **1099**, **1868**) including the human sexual hormone estrone (i.e. **729**), as well as the plant phenylethylamine alkaloid hordenine (i.e. **3499**).

**Table 1:** Bacterial strains and plasmids used in this study.

Strains and plasmids	Relevant characteristics	Reference or source
*Agrobacterium* NT1(pZLR4)	*A. tumefaciens* C58 derivativeexpressing *traR* and *traG-lacZ*, AHL-bioindicator	[42]
*Agrobacterium pTi-donor*	*A. tumefaciens* C58 derivative with pTiC58*ΔaccRΔtraIKm*	[45]
*A. tumefaciens* C58-00	*A. tumefaciens* C58 derivative, cured of its plasmids, recipient strain	Lab collection, CNRS, Gif-sur-Yvette
*Chromobacterium violaceum* CV026	*C. violaceum* ATCC 31532derivative, violacein producer, AHL-bioindicator	[41]
*Escherichia coli* JLD271	K-12 derivative*ΔlacX74sdiA271*::Cam	[21]
*Pseudomonas aeruginosa* PAO1	Wild-type	http://www.pseudomonas.med.ecu.edu/
pβ01	pQF50-derivative, P*_lasB_-lacZ*	[51]
pβ02	pQF50-derivative,P*_rhlA_-lacZ*	[51]
pLPR1	pLP170-derivative, P*_rhlI_-lacZ*	[52]
pPCS223	pLP170-derivative, P*_lasI_-lacZ*	[52]
pPCS1001	pLP170-derivative, P*_lasR_-lacZ*	[53]
pPCS1002	pLP170-derivative,P*_rhlR_-lacZ*	[53]
pAL101	pSB401-derivative, *rhlR^+^rhlI::luxCDABE*	[21]
pAL102	pSB401-derivative, *rhlI::luxCDABE*	[21]
pAL105	pSB401-derivative, *lasR^+^lasI::luxCDABE*	[21]
pAL106	pSB401-derivative, *lasI::luxCDABE*	[21]
pTB4124	pQF50-derivative, P*_aceA_-lacZ*	[20]

### Synthesis of each diastereoisomer of the QSI ID1248

**Figure 2:**
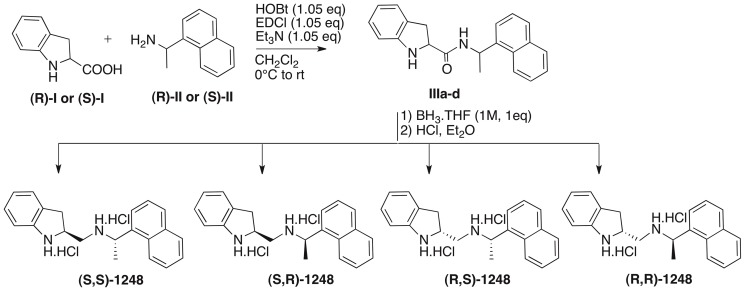
Synthesis of the 4 diastereoisomers of QSI-1248.

Sample **ID1248** being a mixture of 4 stereoisomers, each 4 was synthesized separately and unambiguously starting from the commercially available, optically-active precursors to determine which isomer is the most active (Figure 2). Thus, (*R*)- and (*S*)-indoline-2-carboxylic acid **I** were each coupled with (*R*)- and (*S*)-1-(1-naphthyl)ethylamine **II** using 1-ethyl-3-(3-dimethylaminopropyl)carbodiimide (EDCI) and 1-hydroxybenzotriazole (HOBt) in dichloromethane. The carboxamide bond of each compound formed (**IIIa**-**d**) was then reduced to the amine using borane-THF complex and the products **(** ***S,S*** **)-1248**, **(** ***S,R*** **)-1248**, **(** ***R,S*** **)-1248** and **(** ***R,R*** **)-1248** were isolated as their hydrochloride salts.

### IC_50_ values and bacterial toxicity of the QSIs in A. tumefaciens

**Figure 3:**
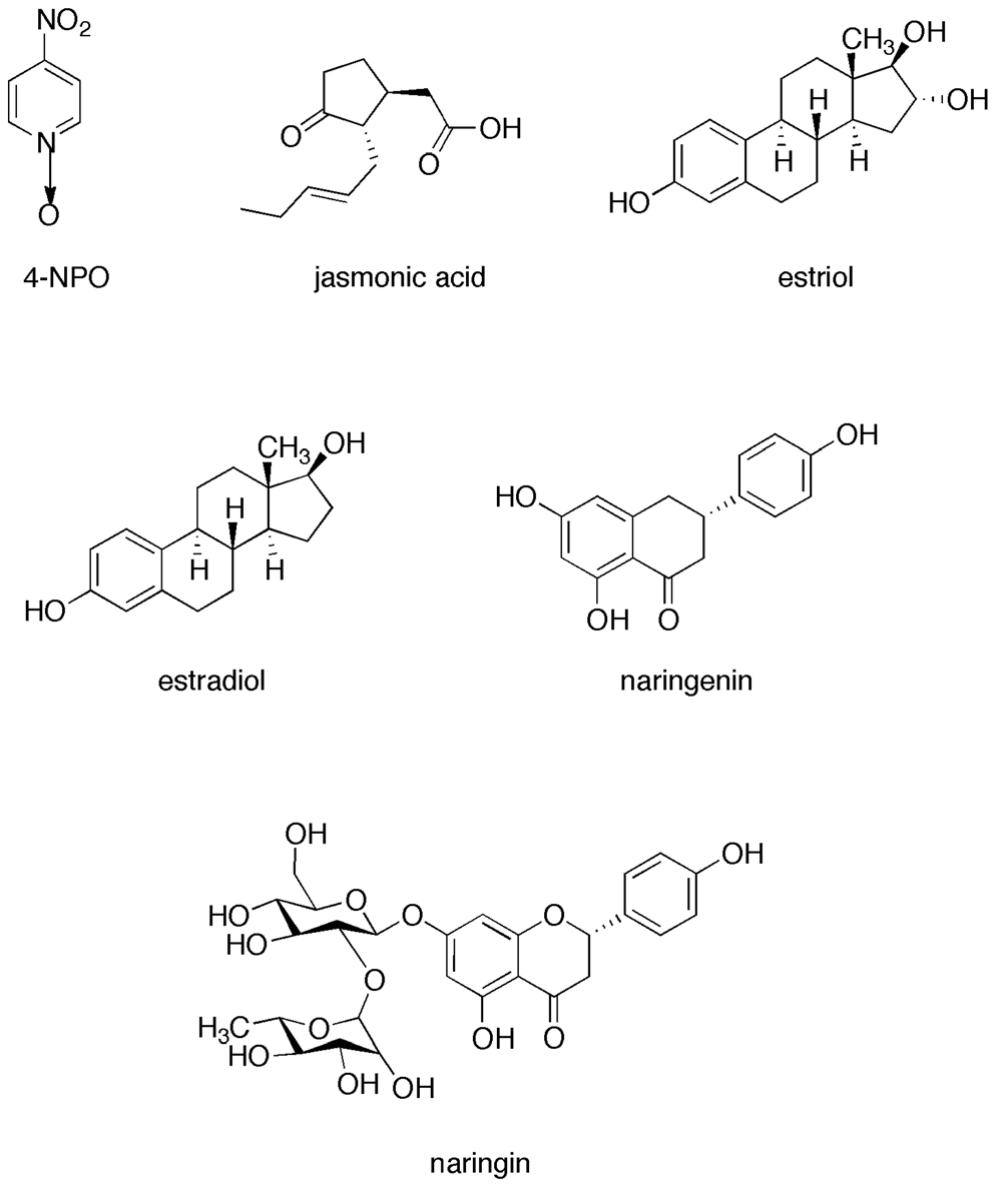
Structure of the additional QSIs and compounds used in this study.

The IC_50_ values of the chemical library QSIs and commercial compounds (Figure 3), such as the hormones estradiol and estriol, the plant-defense signal jasmonic acid, and the QSI-reference 4-nitropyridine-N-oxide (4-NPO) already published by Rasmussen et al [17], were measured using the *A. tumefaciens* bioindicator that expresse the *traG-lacZ* reporter fusion. According to our procedure, the QSI-reference 4-NPO exhibited an IC_50_ of 24 µg/ml (Table 2). Compound **1577** exhibited an IC_50_ value (IC*_50_* = 2.5 µg/ml) lower than that of 4-NPO. The IC_50_ of 10 compounds ranged between 30 and 90 µg/ml (**283**, **729**, **1099**, **1248**, **(S,S)-1248, (S,R)-1248, 3492**, jasmonic acid, estradiol and estriol), while the other IC_50_ values were higher than 100 µg/ml (**14, 15, 937, 1102, 1289, (R,S)-1248, (R,R)-1248 and 3499**). Notably, **(S,R)-1248** exhibited a QSI activity higher than that of the racemic mixture of **1248** and the other diastereoisomers. This stereoselectivity of the inhibitory activity points out the most probable occurrence of a specific interaction of the QSI with the biological target rather than to simple non-specific activity.

**Table 2:** IC50, MIC and MBC values of the tested compounds.

Source	Name	IC_50_ a	MIC^a^	MBC^a^
QSI-reference	4-NPO	24	25	>100
Chemical library	14	>100 (0%)^b^	>100	>100
	15	>100 (19%)	>100	>100
	283	73	>100	>100
	729	75	>100	>100
	937	>100 (15%)	>100	>100
	1099	35	100	>100
	1102	>100 (0%)	>100	>100
	1248	63	>100	>100
	1289	>100 (16%)	>100	>100
	1577	2,5	3	25
	1868	>100 (34%)	12,5	>100
	1949	>100 (38%)	>100	>100
	3028	>100 (32%)	>100	>100
	3492	50	>100	>100
	3499	>100 (19%)	>100	>100
1248-diastereoisomers	(S,S)-1248	90	100	>100
	(S,R)-1248	32	100	100
	(R,S)-1248	>100 (0%)	100	100
	(R,R)-1248	>100 (10%)	100	100
Commercial products	Jasmonic acid	25	>100	>100
	Estradiol	75	>100	>100
	Estriol	50	>100	>100

a values are in µg/ml.

b In brackets, inhibition (%) at 100 µg/ml.

With the exception of compound **1577**, none variation of the cell density (OD_600_) was observed in the IC_50_ determination assay. To know more about bactericidal activity of these compounds, minimum inhibitory concentration (MIC) and minimum bactericidal concentration (MBC) were calculated according to the Andrews' recommendations [18]. The QSI-reference 4-NPO weakly inhibited the growth of *A. tumefaciens* as the MIC value reached 25 µg/ml (Table 2). All the other tested compounds, with the exception of **1577** and **1868** (MIC at 3 and 12.5 µg/ml respectively), exhibited a MIC value equal to or greater than 100 µg/ml, which was the highest concentration tested for determining IC_50_ value and impact of the compounds on QS-regulated plasmid transfer in *A. tumefaciens*. The MBC test confirmed the weak toxicity of the studied QSI because all the MBC values were equal to or higher than 100 µg/ml, with the exception of that of **1577** (MBC at 25 µg/ml).

### QSIs modulate QS-regulated Ti-plasmid transfer in A. tumefaciens

**Figure 4:**
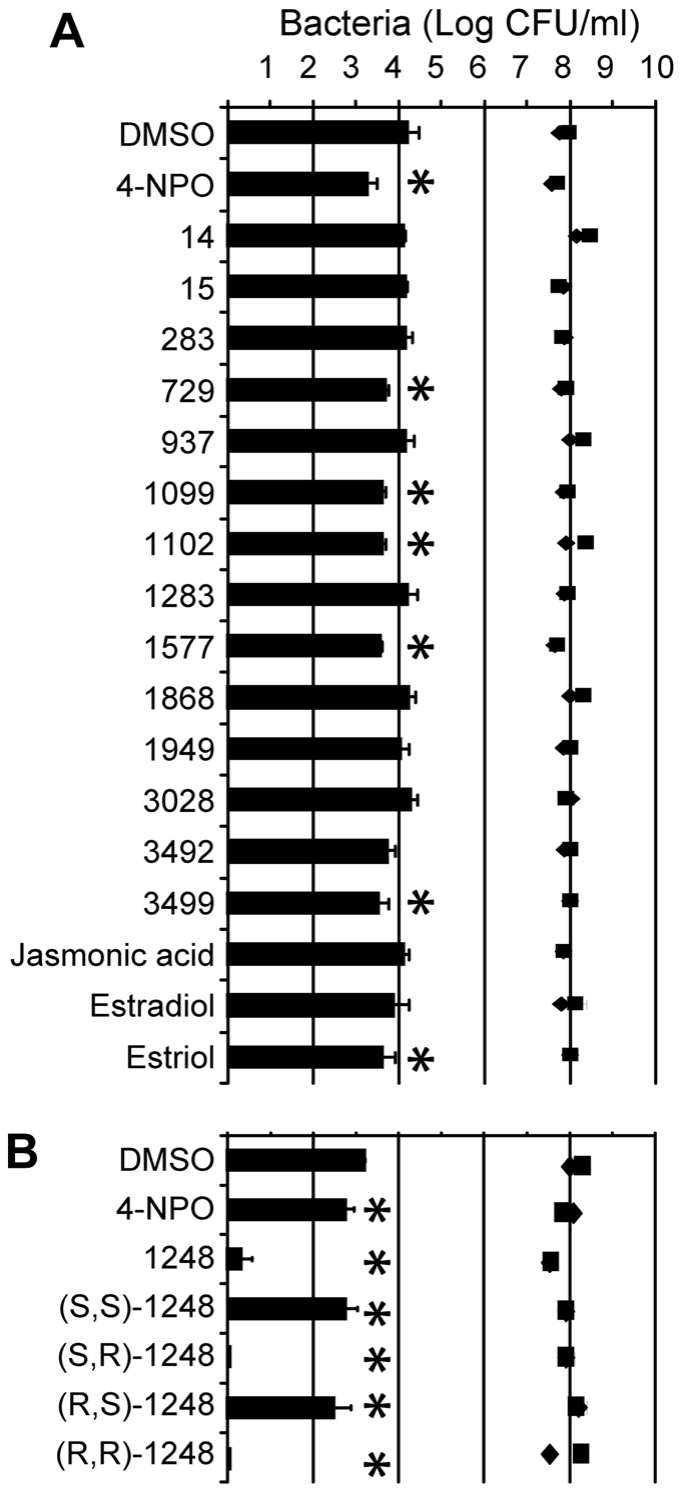
In vitro Ti plasmid transfer frequency in *Agrobacterium.* The Ti plasmid transfer frequencies were measured in the presence of QSI (A) and the four **1248**-diastereoisomeres (B) at 0.1 mg/ml. Histograms represent the cell density of transconjugants (CFU/ml), while black diamonds and squares, those of the donor and recipient strains, respectively. Measurements were performed in quadruplicate and the experiment was repeated twice. The cell densities of transconjugants in the presence of QSI were compared to that of the control in the presence of DMSO with a Mann and Whitney test (α = 0.05). Statistically different values are noted by asterisks.

In *A. tumefaciens*, QS positively regulates horizontal transfer of the virulence plasmid (called Ti plasmid) from a donor strain to a recipient strain. Recipient strains which have received the plasmid are called transconjugants. In this assay, a Ti-plasmid donor and recipient strains were mixed in the presence of AHL (OC8-HSL) and QSI. Resulting transconjugants were counted on rich agar medium supplemented with appropriate antibiotics. When the QSI-reference 4-NPO was added at 0.1 mg/ml, the plasmid transfer efficiency decreased by 10-fold (Figure 4A). Five chemical library-QSIs (**729**, **1099**, **1102, 1577** and **3499**) and estriol were also able to significantly reduce the plasmid transfer frequency by one order of magnitude. In another experiment (Figure 4B), the four **1248**-diastereoisomers were compared. All of them significantly affected horizontal transfer of the Ti plasmid. Noticeably, in conjugation assays, the level of the donor and recipient cells remained at a high level (around 10^8^ CFU/ml, Figure 4), suggesting that the measured decrease in plasmid transfer would not caused by a toxic effect of the tested compounds.

### QSIs modulate QS-signal accumulation in P. aeruginosa

Because the assays with the plant pathogen *A. tumefaciens* highlighted human hormones as QSIs (estrone = **729**, estriol, and estradiol), their QSI-activity was evaluated with the opportunistic pathogen *P. aeruginosa*.

**Figure 5:**
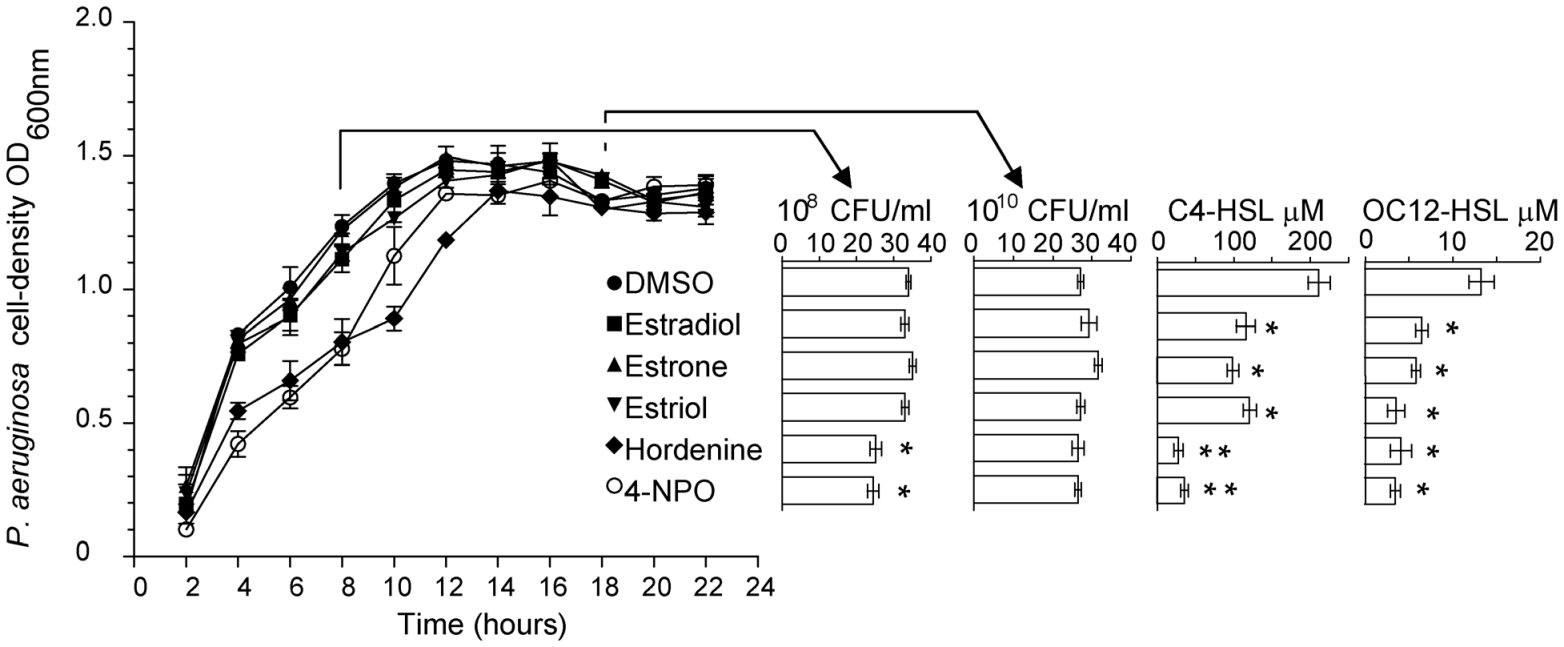
QSIs modulated QS-signal accumulation in *P. aeruginosa.* Growth kinetics (n = 6) of *P. aeruginosa* was measured (OD_600_) in the presence of the QSIs (estradiol, estrone, estriol, hordenine and 4-NPO) at 0.5 mg/ml using DMSO as a negative control. Cell counts were assessed (CFU/ml) at 8- and 18-hour, and C4-HSL and OC12-HSL concentration (µM) were determined in the bacterial cultures at 18-hour. Statistically different values (Student's t test with α = 0.01) are noted by asterisks.

Growth curves of *P. aeruginosa* cells were determined in the presence of the QSIs (Figure 5). The addition of estradiol, estrone and estriol at 0.5 mg/ml did not affect the growth of *P. aeruginosa*. Only hordenine and 4-NPO slightly delayed the growth of *P. aeruginosa*. This was further confirmed by the enumeration of CFU after 8- and 18-hour of incubation. Noticeably, in all cases, the *Pseudomonas* cells reached the same final cell density at the stationary phase. At 18-hour, the concentrations of the AHLs butyrylhomoserine lactone (C4-HSL) and 3-oxo-dodecanoylhomoserine lactone (OC12-HSL), which are produced by *P. aeruginosa*, were quantified using mass spectrometry as described by Vandeputte et al. [19]. The presence of the human hormones estradiol, estrone and estriol, as well as that of hordenine and 4-NPO provoked a reduction of the C4-HSL and OC12-HSL concentrations in the cell cultures, suggesting the QS-signal synthesis and QS-signalling were affected.

### QSIs modulate QS-regulated genes in P. aeruginosa

**Figure 6:**
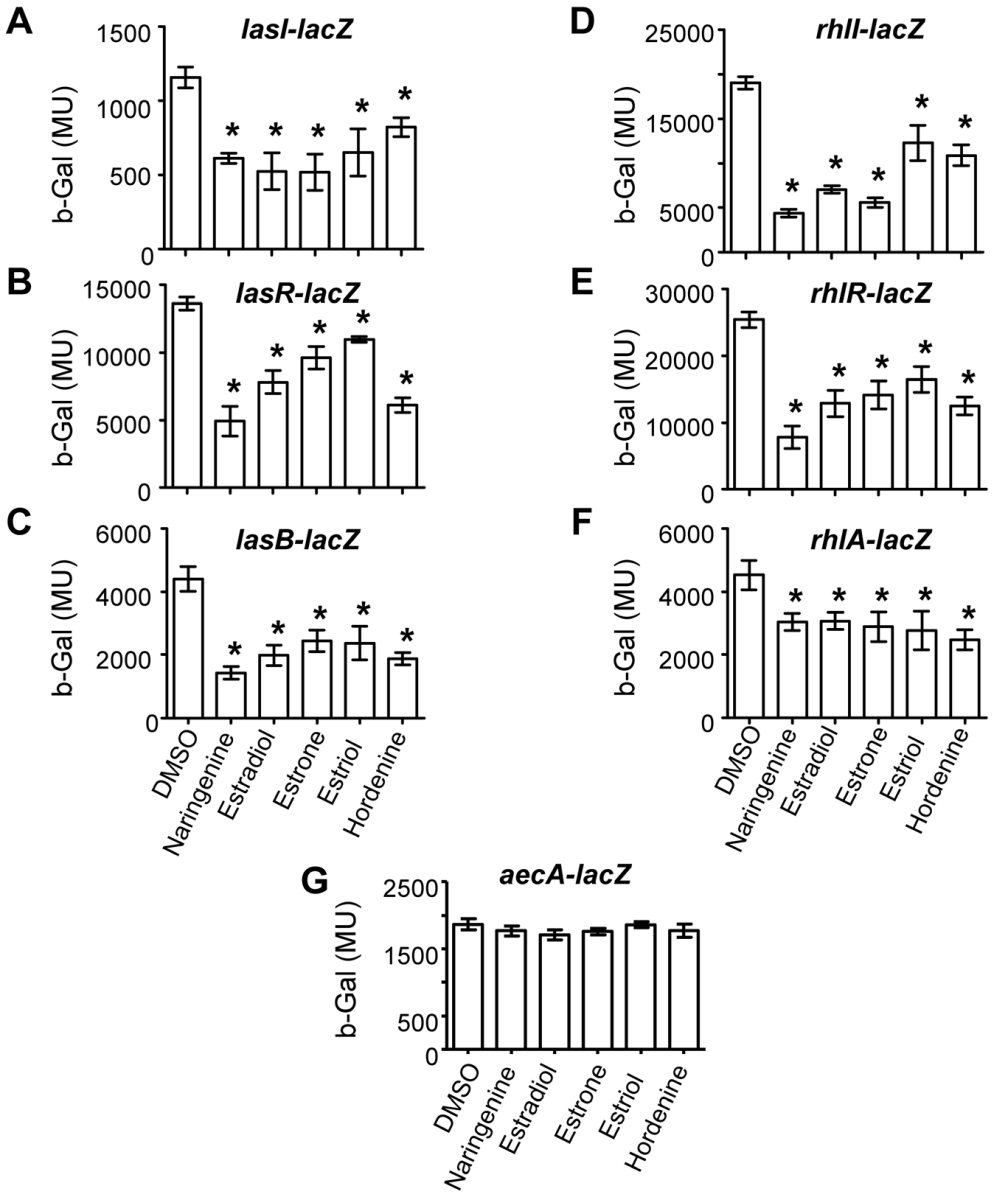
QSIs modulated QS-regulated genes in *P. aeruginosa.* The β-galactosidase (b-Gal) activity in Miller unit (MU) of the transcriptional fusions *lasI-lacZ* (A), *lasR-lacZ* (B), *lasB-lacZ* (C), *rhlI-lacZ* (D), *rhlR-lacZ* (E), *rhlA-lacZ* (F), and *aecA-lacZ*(G) were measured in the presence of estradiol, estrone, estriol, hordenine at 0.5 mg/ml, and naringenin at 1 mg/ml as a positive control and DMSO as a negative control. The statistical significance of each test (n = 5 and three biological replicates) was evaluated by Student's *t* test (i.e. each test was compared with the DMSO condition). Asterisks indicate statistically different data (*p* value of ≤0.01).

The expression of six QS-regulated genes was measured in *P. aeruginosa*: the AHL-synthetase genes *lasI* and *rhlI*, the AHL-sensor genes *lasR* and *rhlR*, and downstream genes *lasB* and *rhlA*, which are regulated by the *las* and *rhl* systems, respectively (Figure 6). The effect of the QSIs was compared to that of naringenin, a known QSI in *P. aeruginosa* [19]. All tested compounds markedly affected the expression of the synthetase genes *lasI* and *rhlI*, though estriol and hordenine had a lower impact on *rhlI* expression than naringenin, estradiol and estrone. Expression of the regulators *lasR* and *rhlR* was also affected, with hordenine being the most and estriol the least potent inhibitors. Other QS-regulated genes, *lasB* and *rhlA*, were also down-regulated in the presence of the tested QSIs. To verify that the drop in *β*-galactosidase activity of the reporter genes was indeed associated with a reduction in QS-related gene expression and not with a general effect on transcription/translation mechanisms, the activity of the *aceA* promoter, the expression of which is not regulated by QS [20] was assessed. The addition of the QSIs did not modify the transcription of the *aceA* gene (Figure 6), indicating that these compounds affected the expression of QS-related genes without affecting the transcription machinery of *P. aeruginosa* PAO1.

### QSIs modulate activity of the QS-signal sensors LasR and RhlR of P. aeruginosa

We determined whether the AHL-binding transcriptional factors LasR and RhlR were impaired in their capacity to activate expression of the QS-regulated genes in the presence of various QSIs. This was achieved by using two *E. coli* bioindicator strains expressing either LasR or RhlR proteins and harboring an appropriate reporter *lux* operon for measuring their transcriptional activity in the presence of OC12-HSL at 100 µM and C4-HSL at 10 µM, respectively [21]. Adding C4-HSL to the pAL101-bioindicator or OC12-HSL to the pAL105-bioindicator induced the expression of the reporter *lux* operon and the consequent production of luminescence. In contrast, only background levels of luminescence were detected in control strains harboring the plasmids pAL102 or pAL106, which lacks the *rhlR* or *lasR* gene, respectively.

**Figure 7:**
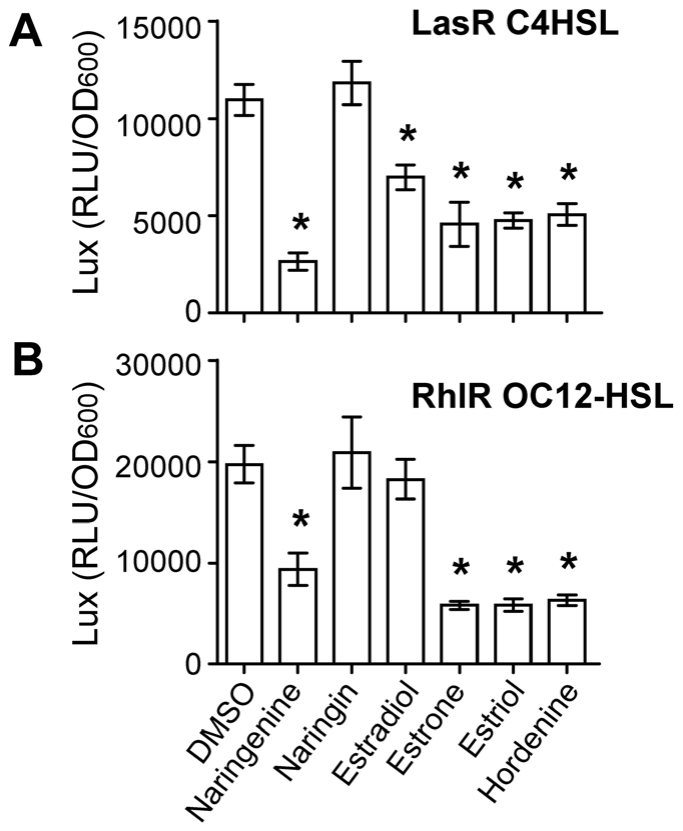
QSIs modulated activity of the AHL-sensors in *P. aeruginosa.* Luminescence of the reporting operon *lux*, expressed in relative light units (RLU/OD_600_), was measured in *E. coli* bioindicator strains harboring the LasR (A) and RhlR (B) AHL-sensing systems of *P. aeruginosa* in the presence of C4-HSL and OC12-HSL, respectively. The QSIs estradiol, estrone, estriol and hordenine were added at 0.1 mg/ml, while naringenin and naringin, added at 0.5 mg/ml, were used as QSI-reference and non-QSI reference, respectively. Each test (n = 6) was compared with the DMSO-condition using Student's *t* test. Asterisks indicate statistically different data (*p* value of ≤0.01).

As shown in Figure 7, both biosensor strains produced less luminescence when naringenin was added to the growth medium as compared with DMSO-treated biosensor cells, indicating that the functioning of the LasR and RhlR proteins was impaired, while the structurally-related compound naringin had no effect. Estradiol affected LasR functionality but not that of RhlR. The other QSIs, estrone, estriol and hordenine, had a significant impact on the perception of both OC12-HSL and C4-HSL by LasR and RhlR, respectively. This observation indicated that the inhibition of the expression of the QS genes in *P. aeruginosa* PAO1 was likely due to a competition between the endogenous AHLs and the tested QSIs to access the AHL-binding site of the LasR and RhlR transcription factors.

### Modeling of the QSI-LasR and QSI-TraR interactions

**Figure 8:**
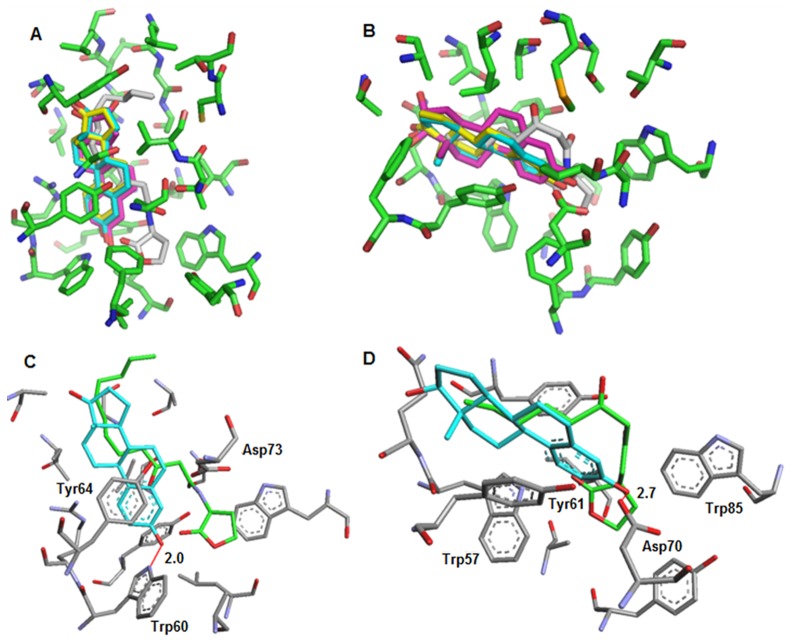
Modeling of interactions between AHL-sensors and human hormones. Superimposed modeling of the overall binding modes of estradiol (cyan), estriol (magenta), estrone (yellow), or AHLs (OC12-HSL or OC8-HSL in gray) within the binding site of LasR (A) and of TraR (B).Superimposed modeling of the simplified binding modes showing interactions between estradiol (selected as an example in cyan) or natural ligands (OC12-HSL or OC8-HSL in green) and binding sites residues of LasR (C) and TraR (D).

In silico docking experiments of estrone, estriol, and estradiol within the binding sites of LasR and TraR were performed. The structure of RhlR is not available and modeling the docking of QSIs could therefore not be achieved. Superimposition of binding models revealed a similar binding mode for the three hormones within the AHL-binding pocket of either LasR or TraR. Notably, the hormone binding modes depicted in Figure 8 were slightly different for LasR and TraR. Careful examination of these binding modes suggested that the saturated rings of these hormones were located in the protein area interacting with the alkyl chain of QS-signals OC12-HSL and OC8-HSL (Figure 8). In the proposed models, the aromatic ring of hormones interacted with a conserved residue in the LuxR family, namely Tyr64 (LasR) or Tyr61 (TraR), while a hydrogen bond links their phenol group with Trp60 of LasR and Asp70 of TraR. Modeling suggests that the LasR/TraR residues, which were involved in the binding of human hormones, were also required for AHL-binding.

## Discussion

This article reports the identification of novel QSIs such as the natural plant compound hordenine and the synthetic indoline-2-carboxamides, and also demonstrates the QSI-activity of the three human sexual hormones that are estrone, estriol and estradiol.

QSIs have been identified in many organisms, plants being the most frequently investigated source of QSI compounds and algae the providers of the most potent ones [1], [5]. Our results revealed the QSI potentiality of alkaloids such as hordenine (**ID 283**), **1248**, and **3492**. Hordenin (CAS# 3595-05-9) is a natural alkaloid of the phenethylamine class exhibiting a widespread occurrence in plants (ornamentals, fruits and vegetables), including those that are used for human and animal consumption [22]–[23]. Following injection, hordenine stimulates the release of norepinephrine in mammals hence acting indirectly as an adrenergic drug [24]–[25]. In the literature, alkaloid compounds have been less frequently reported as acting as QSI than aromatic or polyaromatic compounds [1]. Indeed, solenopsin A, a venom alkaloid produced by the fire ant *Solenopsis invicta*, has been shown to inhibit biofilm formation, pyocyanin and elastase production as well as the expression of QS-regulated genes *lasB*, *rhlI* and *lasI* in *P. aeruginosa* [26]. Peters and co-workers [27] also demonstrated that brominated tryptamine-based alkaloids from *Flustrafoliacea*, a sea bryozoan, inhibit AHL-regulated gene expression using biosensors *P. putida* (pKR-C12), *P. putida* (pAS-C8) and *E. coli* (pSB403) *lasR*, *cepR* and *luxR* coupled to the promoter of *lasB*, *cepI* and *luxI*, respectively.

In this study, the QSI activity of human hormones was supported by complementary features. The pure hormones, especially estriol and estrone, affected expression of the QS-regulated reporter fusion *traG-lacZ* and QS-dependent horizontal transfer of the virulence Ti-plasmid in *A. tumefaciens*. They also decreased the expression of six QS-regulated genes *lasI, lasR, lasB, rhlI, rhlR*, and *rhlA* in *P. aeruginosa*, but none decreased expression of the QS-independent gene *aceA*. Because of the effect on *lasI* and *rhlI*, the AHL concentration was also affected in the presence of the sexual hormones. In agreement with a previous report comparing the effect of steroid hormones on the growth of several pathogens [28], they did not affect the growth of *A. tumefaciens* and *P. aeruginosa* at the concentrations used for describing QSI activity. The sexual hormones act as QSIs at a mM-range concentration which is similar to that of the natural polycyclic QSIs such as catechin and naringenin [19], [29], but higher than that of some other natural and synthetic QSIs which act at a µM range or lower [3], [30]. Our work also revealed that pure hormones affected the QS-regulated reporter gene of *P. aeruginosa* when RhlR or LasR was expressed in *E. coli* in the presence of the appropriate AHL. Moreover, molecular modeling confirmed the competitive hormone-binding capacity of the two AHL-sensors LasR and TraR, suggesting that the AHL-LuxR sensors are targets of the discovered QSIs. This mechanism of action is frequently encountered among QSIs [3]. Such a putative cross-talk between QS and hormonal signalling was hypothesized in prospective reviews by Rumbaugh [31] and Hughes and Sperandio [32] and in a paper reporting docking-type screening of QSIs [33], but, to our knowledge, was never experimentally observed *in vitro* until this report.

Finally, the hypothesis rose about QSI-activity of sexual hormones *in vivo* because the opportunistic pathogen *P. aeruginosa* is detectable in several tissues and organs of hospitalized patients and healthy women, and can thus come into contact with sexual hormones [34]–[36]. A major argument against this hypothesis is that QSI activity of hormones was observed at 2 mM (0.5 mg/ml) while, in serum, concentrations of hormones such as estradiol reach up to 0.4–1.6 nM (100–400 ng/ml) in healthy women and 2–18 nM during fertilizing protocols [37]. However, the debate remains still unclosed because clinical and environmental *Pseudomonas* isolates are known for their capacity to import, bind and biodegrade human hormones, including estrogens, via proteins and pathways that are still poorly-characterized [38]–[40]. These hormone-modifying capabilities would contribute to underestimate the QSI-efficiency of hormones in our *in vitro* assay.

## Materials and Methods

### Instrumentation

Infrared spectra were recorded on a Perkin Elmer Spectrum BX FT-IR spectrometer. Proton (^1^H) and carbon (^13^C) NMR spectra were recorded on Bruker spectrometers: Avance 300 MHz (QNP - ^13^C- probe or Dual ^13^C probe) and Avance 500 MHz (BB0 - ATM probe or BBI - ATM probe). Carbon NMR (^13^C) spectra were recorded at 125 or 75 MHz, using a broadband decoupled mode with the multiplicities obtained using a JMOD or DEPT sequence. NMR experiments were carried out in deuterochloroform (CDCl_3_) or dimethyl sulfoxide (*d_6_*-DMSO), chemical shifts (δ) are reported in parts per million (ppm) with reference to CDCl_3_ (^1^H: 7.24; ^13^C: 77.23). The following abbreviations are used for the proton spectra multiplicities: s: singlet, bs: broad singlet, d: doublet, t: triplet, q: quartet, hept: heptuplet, m: multiplet, br: broad. Coupling constants (J) are reported in Hertz (Hz). Mass spectra were obtained either with a LCT (Micromass) instrument using electrospray ionization (ES), or from a Time of Flight analyzer (ESI-MS) for the high resolution mass spectra (HRMS). The purity and the exact mass were determined for **ID 1949** and **ID 283** with a Waters Acquity liquid chromatograph equipped with a Photodiode Array Detector, an Evaporative Light Scattering Detector and a Triple Quadripole Detector. A reverse-phase HSS T3 column, 2.6 µm, 4.6×100 mm was used for the UPLC work with a mixture acetonitrile/water as the solvent system.

Elemental analyses were performed on a Perkin Elmer CHN 2400 analyzer with detection by catharometry. Thin-layer chromatography was performed on silica gel 60 F254 on aluminium plates (Merck) and visualized under a UVP Mineralight UVLS-28 lamp (254 nm) and with ninhydrin and p-anisaldehyde in ethanol. Flash chromatography was conducted on Merck silica gel 60 (40–63 µm) at medium pressure (300 mbar) or on CombiFlash apparatus (Serlabo Technologies), using standard settings. Reagents and substrates were purchased from Sigma-Aldrich Chemical Company.

### Compounds: General Procedures

Procedure A-Preparation of carboxamides IIIa-d: To a solution of the indolinylcarboxylic acid I (1 eq) in dry methylene chloride (0.3M) at 0°C, was added the amine II (1.05 eq), 1-hydroxybenzotriazole (HOBt, 1.05 eq), 1-ethyl-3-(3-dimethylaminopropyl)carbodiimide (EDCI, 1.05 eq) and triethylamine (1.05 eq). The mixture was stirred at 0°C for 1 h and at room temperature for 5 h. The reaction mixture was quenched with water and extracted with methylene chloride. The organic phase was washed successively with saturated aqueous Na_2_SO_4_ and saturated aqueous NaCl, dried over MgSO_4_, filtered and concentrated under reduce pressure. The residue was purified by flash chromatography on silica gel (elution with heptane/EtOAc 0 to 20%).

Procedure B-Reduction of carboxamides IIIa-d: To a solution of the amide derivatives IIIa-d (1 eq) in dry THF (0.03M) at 0°C, was added BH_3_.THF (1M, 1 eq) and the mixture was stirred at reflux for 16 h. The reaction mixture was cooled to 0°C, acidified with aqueous HCl (2M) and refluxed for an additional 30 min. The mixture was then extracted with methylene chloride and the organic phase was washed successively with saturated aqueous Na_2_CO_3_, saturated aqueous NaCl, dried over MgSO_4_, filtered and concentrated under reduce pressure. The residue was purified by flash chromatography on silica gel (elution with heptane/EtOAc 0 to 30%). The pure amine product was dissolved in diethyl ether, a solution of HCl in diethyl ether (2M) was added and the precipitated hydrochloride salt was collected by filtration, washed with diethyl ether and dried under vacuum.

#### (S)-N-((S)-1-(naphthalen-1-yl)ethyl)indoline-2-carboxamide (IIIa)

Following general procedure A using (S)-indoline-2-carboxylic acid (0.61 mmol, 100 mg), (S)-1-(1-naphthyl)ethylamine (0.64 mmol, 103 µL), HOBt (0.64 mmol, 86 mg), EDCl (0.64 mmol, 123 mg) and Et_3_N (0.64 mmol, 46 µL) in methylenechloride (2 mL), IIIa was obtained as a white solid (182 mg, 94%). ^1^H NMR (CDCl_3_): 8.17 (d, J = 8.3 Hz, 1H), 7.91 (d, J = 7.3 Hz, 1H), 7.84 (d, J = 8.3 Hz, 1H), 7.60−7.46 (m, 4H), 7.36−7.34 (m, 1H), 7.14 (d, J = 7.3 Hz, 1H), 7.07 (t, J = 8.3 Hz, 1H), 6.84 (td, J = 7.3, 1.0 Hz, 1H), 6.65 (d, J = 7.3 Hz, 1H), 6.04−5.95 (m, 1H), 4.46−4.40 (m, 1H), 4.05 (bs, 1H), 3.69−3.60 (m, 1H), 3.19−3.11 (m, 1H), 1.68 (d, J = 7.0 Hz, 3H); ^13^C NMR (CDCl_3_): 172.6, 149.5, 138.2, 134.0, 131.2, 128.8, 128.4, 128.0, 127.6, 125.9, 125.3, 124.8, 124.3, 123.4, 122.7, 120.6, 110.8, 61.4, 44.0, 35.6, 20.7; IR (neat) 3298, 2900, 1651, 1608, 1512, 1484, 1467, 1245; MS (ESI): [M+H] m/z 317.2.

#### (S)-N-((R)-1-(naphthalen-1-yl)ethyl)indoline-2-carboxamide (IIIb)

Following general procedure A using (S)-indoline-2-carboxylic acid (0.61 mmol, 100 mg), (R)-1-(1-naphthyl)ethylamine (0.64 mmol, 103 µL), HOBt (0.64 mmol, 86 mg), EDCl (0.64 mmol, 123 mg) and Et_3_N (0. mmol, 46 µL) in methylenechloride (2 mL), IIIb was obtained as a white solid (105 mg, 54%). ^1^H NMR (CDCl_3_): 8.12 (d, J = 8.3 Hz, 1H), 7.86 (d, J = 7.6 Hz, 1H), 7.79 (d, J = 8.3 Hz, 1H), 7.56−7.40 (m, 4H), 7.36−7.31 (m, 1H), 7.04 (t, J = 7.6 Hz, 2H), 6.78 (td, J = 7.3, 1.2 Hz, 1H), 6.69 (d, J = 7.6 Hz, 1H), 6.05−5.95 (m, 1H), 4.53−4.47 (m, 1H), 4.15 (bs, 1H), 3.60−3.49 (m, 1H), 2.99−2.91 (m, 1H), 1.72 (d, J = 6.8 Hz, 3H); ^13^C NMR (CDCl_3_): 172.4, 149.5, 138.3, 133.9, 131.1, 128.8, 128.3, 127.9, 127.6, 126.4, 125.8, 125.2, 124.8, 123.3, 122.3, 120.6, 110.8, 62.0, 44.1, 35.5, 21.2; IR (neat) 3298, 2900, 1651, 1608, 1512, 1484, 1467, 1245; MS (ESI): [M+H] m/z 317.2.

#### (R)-N-((S)-1-(naphthalen-1-yl)ethyl)indoline-2-carboxamide (IIIc)

Following general procedure A using (R)-indoline-2-carboxylic acid (0.61 mmol, 100 mg), (S)-1-(1-naphthyl)ethylamine (0.64 mmol, 103 µL), HOBt (0.64 mmol, 86 mg), EDCl (0.64 mmol, 123 mg) and Et_3_N (0.64 mmol, 46 µL) in methylenechloride (2 mL), IIIc was obtained as a white solid (141 mg, 73%). ^1^H NMR (CDCl_3_): 8.12 (d, J = 8.3 Hz, 1H), 7.86 (d, J = 7.6 Hz, 1H), 7.80 (d, J = 8.3 Hz, 1H), 7.56−7.40 (m, 4H), 7.36−7.31 (m, 1H), 7.04 (t, J = 7.6 Hz, 2H), 6.79 (td, J = 7.3, 1.2 Hz, 1H), 6.69 (d, J = 7.6 Hz, 1H), 6.05−5.95 (m, 1H), 4.50−4.48 (m, 1H), 4.15 (bs, 1H), 3.61−3.49 (m, 1H), 3.00−2.87 (m, 1H), 1.72 (d, J = 6.8 Hz, 3H); ^13^C NMR (CDCl_3_): 172.4, 149.5, 138.3, 133.9, 131.1, 128.8, 128.3, 127.9, 127.6, 126.4, 125.8, 125.2, 124.8, 123.3, 122.3, 120.6, 110.8, 62.0, 44.1, 35.5, 21.2; IR (neat) 3352, 2900, 1651, 1608, 1511, 1484, 1466, 1244; MS (ESI): [M+H] m/z 317.2.

#### (R)-N-((R)-1-(naphthalen-1-yl)ethyl)indoline-2-carboxamide (IIId)

Following general procedure A using (R)-indoline-2-carboxylic acid (0.61 mmol, 100 mg), (R)-(1-(1-naphthyl)ethylamine (0.64 mmol, 103 µL), HOBt (0.64 mmol, 86 mg), EDCl (0.64 mmol, 123 mg) and Et_3_N (0.64 mmol, 46 µL) in methylenechloride (2 mL), IIId was obtained as a white solid (116 mg, 60%). ^1^H NMR (CDCl_3_): 8.12 (d, J = 8.3 Hz, 1H), 7.86 (d, J = 7.6 Hz, 1H), 7.80 (d, J = 8.3 Hz, 1H), 7.56−7.40 (m, 4H), 7.36−7.31 (m, 1H), 7.04 (t, J = 7.6 Hz, 2H), 6.79 (td, J = 7.3, 1.2 Hz, 1H), 6.69 (d, J = 7.6 Hz, 1H), 6.05−5.95 (m, 1H), 4.50−4.48 (m, 1H), 4.01 (bs, 1H), 3.61−3.49 (m, 1H), 3.00−2.87 (m, 1H), 1.68 (d, J = 6.8 Hz, 3H); ^13^C NMR (CDCl_3_): 172.6, 149.5, 138.3, 133.9, 131.1, 128.8, 128.3, 127.9, 127.6, 126.4, 125.8, 125.2, 124.8, 123.3, 122.3, 120.6, 110.8, 61.4, 44.0, 35.6, 20.7; IR (neat) 3352, 2925, 2900, 1651, 1608, 1511, 1484, 1466, 1246; MS (ESI): [M+H] m/z 317.2.

#### (S)-N-((S)-indolin-2-ylmethyl)-1-(naphthalen-1-yl)ethanamine ((S,S)-1248)

Following the general procedure B using amide IIIa (0.25 mmol, 80 mg), BH_3_.THF (1M, 1.21 mL) in dry THF (8 mL) afforded ((S,S)-1248 (42 mg, 55%) as a colorless oil. ^1^H NMR (CDCl_3_): 8.19 (dd, J = 7.1, 2.1 Hz, 1H), 7.87 (dd, J = 7.1, 2.1 Hz, 1H), 7.76 (d, J = 8.1 Hz, 1H), 7.64 (d, J = 8.1 Hz, 1H), 7.54−7.45 (m, 3H), 7.06−6.98 (m, 2H), 6.66 (t, J = 7.1 Hz, 1H), 6.62 (d, J = 8.1 Hz, 1H), 4.75−4.60 (m, 1H), 3.94−3.84 (m, 1H), 3.09 (dd, J = 15.9, 8.9 Hz, 1H), 2.72−2.65 (m, 3H), 1.51 (d, J = 6.7 Hz, 3H); ^13^C NMR (CDCl_3_): 150.8, 141.3, 134.0, 131.3, 129.0, 128.7, 127.3, 127.2, 125.9, 125.7, 125.4, 124.8, 123.0, 122.7, 118.7, 109.7, 59.3, 54.0, 53.3, 34.1, 23.6; IR (neat) 3361, 2973, 2926, 2853, 1609, 1485, 1465, 1115; HRMS (ESI): calcd. for C_21_H_23_N_2_ [M+H] m/z 303.1861, found m/z 303.1863. Hydrochloride derivative: Anal. Calcd. C, 67.20; H, 6.45; N, 7.46. Found C, 62.07; H, 6.22; N, 6.76.

#### (R)-N-((S)-indolin-2-ylmethyl)-1-(naphthalen-1-yl)ethanamine ((S,R)-1248)

Following general procedure B using amide IIIb (0.30 mmol, 95 mg), BH3.THF (1M, 1.45 mL) in dry THF afforded ((R,S)-1248 (74 mg, 81%) as a colorless oil. ^1^H NMR (CDCl_3_): 8.31 (d, J = 8.5 Hz, 1H), 7.97 (d, J = 7.2 Hz, 1H), 7.83 (d, J = 8.5 Hz, 1H), 7.74 (d, J = 7.2 Hz, 1H), 7.64−7.53 (m, 3H), 7.15−7.08 (m, 2H), 6.78 (t, J = 7.2 Hz, 1H), 6.68 (d, J = 7.8 Hz, 1H), 4.77−4.70 (m, 1H), 4.04−3.92 (m, 1H), 3.20−3.12 (m, 1H), 2.86−2.78 (m, 2H), 2.72−2.66 (m, 1H), 1.62 (d, J = 6.5 Hz, 3H); ^13^C NMR (CDCl_3_): 150.9, 140.9, 134.1, 131.4, 129.1, 128.7, 127.3, 127.0, 125.8, 125.8, 125.4, 124.9, 123.1, 122.9, 118.7, 109.7, 59.1, 53.9, 52.8, 33.9, 23.8; IR (neat) 3363, 2973, 2927, 2864, 1608, 1484, 1464, 1247, 1114; HRMS (ESI): calcd. for C_21_H_23_N_2_ [M+H] m/z 303.1861, found m/z 303.1873. Hydrochloride derivative: Anal. Calcd. C, 67.20; H, 6.45; N, 7.46. Found C, 67.27; H, 6.78; N, 7.25.

#### (S)-N-((R)-indolin-2-ylmethyl)-1-(naphthalen-1-yl)ethanamine ((R,S)-1248)

Following general procedure B using amide IIIc (0.35 mmol, 110 mg), BH_3_.THF (1M, 1.70 mL) in dry THF (10 mL) afforded (S,R)-1248 (76 mg, 72%) as a colorless oil. ^1^H NMR (CDCl_3_): 8.31 (d, J = 8.5 Hz, 1H), 7.96 (d, J = 7.2 Hz, 1H), 7.83 (d, J = 8.5 Hz, 1H), 7.74 (d, J = 7.2 Hz, 1H), 7.64−7.53 (m, 3H), 7.15−7.08 (m, 2H), 6.78 (t, J = 7.2 Hz, 1H), 6.68 (d, J = 7.8 Hz, 1H), 4.77−4.70 (m, 1H), 4.04−3.92 (m, 1H), 3.20−3.12 (m, 1H), 2.86−2.78 (m, 2H), 2.72−2.66 (m, 1H), 1.62 (d, J = 6.5 Hz, 3H); ^13^C NMR (CDCl_3_): 150.9, 141.0, 134.1, 131.4, 129.1, 128.7, 127.3, 127.0, 125.8, 125.8, 125.4, 124.9, 123.1, 122.9, 118.7, 109.7, 59.1, 53.9, 52.8, 33.9, 23.8; IR (neat) 3364, 2973, 2927, 2863, 1609, 1484, 1464, 1247, 1114; HRMS (ESI): calcd. for C_21_H_23_N_2_ [M+H] m/z 303.1861, found m/z 303.1862. Hydrochloride derivative: Anal. Calcd. C, 67.20; H, 6.45; N, 7.46. Found C, 68.78; H, 6.78; N, 7.20.

#### (R)-N-((R)-indolin-2-ylmethyl)-1-(naphthalen-1-yl)ethanamine ((R,R)-1248)

Following general procedure B using amide IIId (0.35 mmol, 110 mg), BH_3_.THF (1M, 1.70 mL) in dry THF (10 mL) afforded (R,R)-1248 (76 mg, 72%) as a colorless oil. ^1^H NMR (CDCl_3_): 8.27 (d, J = 8.3 Hz, 1H), 7.96 (d, J = 7.1 Hz, 1H), 7.83 (d, J = 8.3 Hz, 1H), 7.72 (d, J = 7.1 Hz, 1H), 7.62−7.53 (m, 3H), 7.14−7.07 (m, 2H), 6.77 (t, J = 7.1 Hz, 1H), 6.69 (d, J = 7.1 Hz, 1H), 4.71 (q, J = 7.1 Hz, 1H), 4.00−3.90 (m, 1H), 3.20−3.12 (m, 1H), 2.79−2.72 (m, 3H), 1.59 (d, J = 6.7 Hz, 3H); ^13^C NMR (CDCl_3_): 150.8, 141.4, 134.1, 131.3, 129.1, 128.8, 127.3, 127.0, 125.9, 125.7, 125.4, 124.9, 123.1, 122.9, 118.7, 109.8, 59.3, 54.0, 53.4, 34.1, 23.7; IR (neat) 3364, 2973, 2927, 2863, 1609, 1484, 1464, 1247, 1114; HRMS (ESI): calcd. for C_21_H_23_N_2_ [M+H] m/z 303.1861, found m/z 303.1867. Hydrochloride derivative: Anal. Calcd. C, 67.20; H, 6.45; N, 7.46. Found C, 67.64; H, 6.91; N, 7.10.

#### 2,3,11a-Trimethyl-2,3,3a,4,5,5a,5b,6,8,9,10,11,11a,11b,12,13-hexadecahydro-1H-2-aza-pentaleno[1,6a-a]phenanthren-9-ylamine hydrochloride or dihydroconaminehydrochloride ID 283

^1^H NMR (*d_6_*-DMSO): 0.65-0.70 (1H, m, H_9_), 0.72 (3H, s, H_20_), 0.97–1.00 (3H, m, H_1a_ H_4b_ H_12b_), 1.11–1.19 (4H, m, H_8_ H_10_ H_14_), 1.20–1.24 (1H, m, H_3a_), 1.28 (3H, d, J = 6.3 Hz, H_22_), 1.34–1.45 (4H, m, H_7_ H_11a_ H_15a_), 1.50–1.54 (1H, m, H_16b_), 1.57–1.68 (3H, m, H_4b_ H_11b_ H_12b_), 1.70–1.76 (1H, m, H_15b_), 1.81–1.87 (1H, m, H_16a_), 1.99–2.03 (1H, m, H_3b_), 2.16–2.22 (2H, m, H_4_), 2.69 (3H, s, H_21_), 2.86–3.01 (1H, m, H_2_), 3.35–3.40 (2H, m, H_19_), 3.45–3.59 (1H, m, H_18_), 8.10 (2H, s, NH_2_). ^13^C NMR(*d_6_-*DMSO): 11.2 (1C, C_22_), 11.7 (1C, C_20_), 21.3 (1C, C_12_), 21.7 (1C, C_16_),25.4 (1C, C_11_), 25.7 (1C, C_15_), 27.7 (1C, C_8_), 31.4 (1C, C_4_), 32.1 (1C, C_7_), 35.0 (1C, C_5_), 35.9 (2C, C_1_ C_3_),36.9 (1C, C_14_),39.3 (1C, C_31_),43.8 (1C, C_10_), 49.3 (1C, C_2_), 51.3 (1C, C_13_),51.7 (1C, C_17_), 52.3 (1C, C_9_), 53.8 (1C, C_6_), 59.6 (1C, C_19_), 64.6 (1C, C_18_). MS (ESI, m/z)331.3 [M+H]^+^.

#### 3-Hydroxy-13-methyl-6,7,8,9,11,12,13,14,15,17-decahydro-cyclopenta[a] phenanthren-16-one ID 729

^1^H NMR (*d_6_*-DMSO): 0.84 (3H, s, H_18_), 1.30–1.45 (3H, m, H_8a_ H_9_ H_11a_), 1.58 (1H, dt, J_1_ = 3.3 Hz, J_2_ = 12.3 Hz, H_12a_), 1.72–1.79 (2H, m, H_8b_ H_14_), 1.85 (1H, dt, J_1_ = 3.3 Hz, J_2_ = 12.3 Hz, H_12b_), 1.93–1.99 (1H, m, H_15a_), 2.03–2.06(1H, m, H_17_), 2.12–2.16 (1H, m, H_15b_), 2.27–2.33 (2H, m, H_10_ H_11b_), 2.71–2.76 (2H, m, H_7_), 6.42 (1H, d, J = 2.4 Hz, H_1_), 6.51 (1H, dd, J_1_ = 2.4 Hz, J_2_ = 8.4 Hz, H_3_), 7.4 (1H, d, J = 8.7 Hz, H_4_), 8.99 (1H, s, OH). ^13^C NMR(*d_6_*-DMSO):17.8 (1C, C_18_), 25.8, (1C, C_7_), 27.6 (1C, C_8_), 29.0 (1C, C_11_), 37.3 (1C, C_12_), 37.7 (1C, C_14_), 38.3 (1C, C_15_), 38.8 (1C, C_13_), 43.1 (1C, C_10_), 49.7 (1C, C_9_), 55.3 (1C, C_17_), 112.7, (1C, C_3_),114.91 (1C, C_1_), 125.77 (1C, C_4_), 130.11 (1C, C_5_), 136.9 (1C, C_6_), 154.9 (1C, C_2_), 217.1 (1C, C_16_). MS (IE (70 eV), m/z)341.2 [M]^+^.

#### 5-Methyl-1-(2-phenylsulfanyl-ethoxymethyl)-1H-pyrimidine-2,4-dione ID 1949

^1^H NMR (*d_6_*-DMSO), 1.75 (3H, s, H_18_), 3.13 (2H, t, J = 6 Hz, H_10_), 3.64 (2H, t, J = 6 Hz, H_9_), 5.05 (2H, s, H_7_), 7.16–7.733 (5H, m, Ar), 7.54 (1H, s, H_6_),11.29 (1H, s, NH). ^13^C NMR(*d_6_*-DMSO): 11.8 (1C, C_18_), 31.8 (1C, C_10_), 66.83 (1C, C_9_), 75.9 (1C, C_7_), 110.00 (1C, C_5_), 125.8 (1C, C_15_), 128.2 (2C, C_14_ C_16_), 128.9 (2C, C_13_ C_17_), 136.0 (1C, C_12_), 140.3 (1C, C_6_), 152.0 (1C, C_2_), 166.0 (1C, C_4_). MS(ESI, m/z)393.9 [M+H]^+^.

### QS-bioindicators

Two bacterial QS-signal biosensors were used. *Chromobacterium violaceum* CV026 [41] was grown in Luria-Bertani modified (LBm) medium, in which the NaCl concentration was 5 g/l instead of 10 g/l. *Agrobacterium tumefaciens* NT1(pZLR4) [42] was grown in AB minimal medium [43] supplemented with mannitol (0.2%). After supplementation with appropriate AHL, each of the QS-biosensors expresses a reporting activity, which is the production of the pigment violacein in *C. violaceum* CV026 and that of β-galactosidase from the transcriptional fusion *traG::lacZ* in *A. tumefaciens* NT1(pZLR4).

### Library screening for QSI

When the screening was performed, the chemical library of the *Institut de Chimie des Substances Naturelles* (ICSN, Gif-sur-Yvette, France) contained more than 3500 synthetic and natural compounds, which were individually dissolved in dimethylsulfoxide (DMSO) at 1mg/ml and stored in 96 microwell plates. A first screening was performed by mixing biosensor CV026 with the AHL hexanoylhomoserine lactone (C6-HSL) at 0.5 µM and the tested compounds at 50 µg/ml. Final volume was adjusted to 100 µl with a synthetic medium (10% LBm and 0.4% sucrose). After 24 hours of incubation at 30°C, the presence or absence of violacein pigment was quoted by visual reading. A second screening was performed using biosensor *A. tumefaciens* NT1(pZLR4), the AHL octanoylhomoserine lactone (C8-HSL) at 10 nM and the tested molecules at 5 µg/ml. Final volume was adjusted to 100 µl by the addition of AB minimal medium supplemented with mannitol (0.2%) and TY medium (10%). After 4 hours of incubation at 30°C, β-galactosidase activity was measured in *A. tumefaciens* cultures as previously described [44]. In the two screenings, 4-nitropyridine-N-oxide (4-NPO) was used as a QSI reference [17].

### Measurement of IC_50_, MIC and MBC values of the QSI

To determine half maximal inhibitory concentration (IC*_50_*) of QSI, β-galactosidase activity of the *Agrobacterium* biosensor was measured, as described in the screening protocol, in the presence of AHL and QSI, which were introduced at final concentrations ranging from 1.5 µg/ml to 100 µg/ml. Aside from the effect of QSI on QS regulation, the toxicity of these compounds was tested on bacterial cells by measuring two parameters [18]: the minimal inhibitory concentration (MIC) and minimal bactericidal concentration (MBC). MIC, which is the lowest concentration of QSI (µg/ml) inhibiting any visible culture after 36 h of incubation at 30°C, was estimated by culturing 10^5^ CFU/ml of the *Agrobacterium* biosensor cells in the presence of different concentrations of the QSI. MBC, which is the lowest concentration of QSI (µg/ml) that results in a 99.9% reduction of the initial bacterial population (10^5^ CFU/ml) after 36 h of incubation at 30°C in the presence of different concentrations of QSI, was estimated by plating 100 µL of the *Agrobacterium* cultures on agar LBm plates. After an incubation of 48 h at 30°C, CFU were counted and the MBC values were calculated.

### QS-regulated plasmid-transfer in A. tumefaciens

In *A. tumefaciens*, the transfer of the Ti-plasmid from a donor cell to a recipient one is controlled by QS. QSI were evaluated for their capacity to reduce plasmid transfer frequency in *Agrobacterium*. In the plasmid transfer assay, two modified *Agrobacterium* strains were used: a Ti-plasmid donor, which requires exogenous AHL to transfer a kanamycin-resistant (Km^r^) Ti-plasmid [45], and a rifampicin-resistant (Rif^r^) recipient strain, which is free of Ti-plasmid. Overnight cultures of the donor and recipient strains were mixed with 500 pM of oxo-octanoylhomoserine lactone (OC8-HSL) and 0.1 mg/ml of QSI in LBm medium. Each combination was repeated 4 times in microwell plates. After 72 hours of incubation at 24°C, the recipient and donor cells, as well as recipient cell which had acquired the Ti-plasmid from the donors, were counted on LBm agar plates supplemented with the antibiotics Km and Rif.

### QS-regulated genes in Pseudomonas aeruginosa and AHLs quantification

QS gene transcription in *P. aeruginosa* PAO1 was monitored using PAO1 strains carrying various gene promoters fused to a *lacZ* reporter gene, as described in Vandeputte et al., 2010 and Table 1. PAO1 reporter strains were prepared according to Vandeputte et al. [29]. Briefly, 18-hours-old liquid cultures (50 µl) were diluted in order to obtain a starting OD_600 nm_ comprised between 0.02 and 0.03 in fresh LB medium supplemented with 50 mM MOPS pH 7.0 (1 ml), carbenicillin (300 µg/ml) and 10 µl of the QSIs to be tested (OD were measured using a SpectraMax M2 device from Molecular Devices). Test and control QSIs were diluted in 100% DMSO (resulting after addition to the growth medium in a 1% final concentration of DMSO). Cultures were incubated for 18 hours at 37°C with agitation. After incubation, cell densities were assessed spectrophotometrically (OD_600 nm_) and *β*-galactosidase assays were performed using the substrate *o*-nitrophenyl-*β*-D-galactopyranoside as described previously [19], [29]. Promoterless-lacZ fusions were used as controls. AHLs were quantified as described in Vandeputte et al. [19]. All tests were performed in quintuplicates and three biological repetitions. The statistical significance of each test was evaluated by conducting Student's *t* tests and two-way ANOVA combined with the Tukey post-analysis test using the GraphPad Prism software and *p* values≤0.01 were considered significant.

### Heterologous expression of the *Pseudomonas QS-receptors* in *E. coli*

*Escherichia coli* JLD271 biosensor strains harboring LasR- and RhlR-based plasmids pAL105 and pAL101 and control plasmids pAL106 (LasR^-^) and pAL102 (RhlR^−^) (Table 1) were prepared according to Vandeputte et al. [19]. Briefly, these strains were grown in LB medium supplemented with tetracycline (10 µg/ml) and chloramphenicol (25 µg/ml) for 24 h [21]. Then 50-µl portions of the cultures were subcultured in 1 ml of LB medium (the starting OD_600_ ranged between 0.02 and 0.025 corresponding to 5.10^6^ CFU) supplemented with 10 µl of DMSO (1% [vol/vol] final), 10 µl of naringenin or naringin dissolved in DMSO (2 mM, final concentration), or 10 µl of the molecule to be tested. To induce the expression of the *lux* operon, 0, 1, 10 or 100 mM of C4-HSL was added to pAL101 and pAL102, while OC12-HSL was added to pAL105 and pAL106. After incubation for 2 h at 37°C with agitation (175 rpm), 200 µl of culture was transferred to 96-well OptiPlate-96 F plates from Perkin-Elmer, and the luminescence of each sample was measured by using a TopCount NXT device from Perkin-Elmer. The LasR(pAL106) and RhlR(pAL102) biosensors were used for background subtraction, and the OD_600_ values were measured to account for the differences in cell density. All experiments were performed in six replicates. The statistical significance of each test was evaluated by conducting Student's *t* tests using the GraphPad Prism software, and *p* values ≤0.05 were considered significant. Naringenin (4′,5,7-trihydroxyflavanone), naringin (4′,5,7-trihydroxyflavanone 7-rhamnoglucoside), the AHLs OC12-HSL and C4-HSL were purchased from Sigma-Aldrich and dissolved freshly in 100% DMSO before use. Tested QSIs were dissolved in 100% DMSO before use.

### Modeling of the interactions between QSIs and QS-receptors

PDB codes of the estradiol, estriol and estrone were respectively 2YJA (name EST), 1×8 V (name ESL) and 3HM1 (name J3Z) [46]–[47]. Docking experiments were performed on each hormone using Arguslab [48] with X-ray protein structures of LasR (pdb code 2UV0) [49] and TraR (pdb code1L3L) [50]. The docking engine GA (genetic algorithm) was employed with default parameters using a 15 Å docking box centered on natural ligands (OC12-HSL and OC8-HSL). Docking results for each protein were then superimposed with PyMol(http://www.pymol.org/) to generate figures presented in Figure 8 A and B (top). Simplified binding modes (Figure 8 C and D) were constructed with Arguslab using the docking results of estradiol (taken as example) within LasR and TraR by hiding some binding site residues. Hydrogen bonds were assigned within a distance of 3.0 Å.
